# Lipidomic UPLC-MS/MS Profiles of Normal-Appearing White Matter Differentiate Primary and Secondary Progressive Multiple Sclerosis

**DOI:** 10.3390/metabo10090366

**Published:** 2020-09-08

**Authors:** Petros Pousinis, Ines R. Ramos, M. Nicola Woodroofe, Laura M. Cole

**Affiliations:** 1 Biomolecular Sciences Research Centre, Faculty of Health, Wellbeing and Life Sciences, Sheffield Hallam University, Sheffield S1 1WB, UK; pepousinis@chem.auth.gr (P.P.); nw6916@exchange.shu.ac.uk (M.N.W.); 2 Leicester School of Allied Health Sciences, Faculty of Health and Life Science, De Montfort University Leicester, Leicester LE1 9BH, UK; ines.martinsramos@dmu.ac.uk

**Keywords:** multiple sclerosis, progression, lipidomics, neurodegeneration, glycerophospholipids

## Abstract

Multiple sclerosis (MS) is a neurodegenerative inflammatory disease where an autoimmune response to components of the central nervous system leads to a loss of myelin and subsequent neurological deterioration. People with MS can develop primary or secondary progressive disease (PPMS, SPMS) and differentiation of the specific differences in the pathogenesis of these two courses, at the molecular level, is currently unclear. Recently, lipidomics studies using human biofluids, mainly plasma and cerebrospinal fluid, have highlighted a possible role for lipids in the initiation and progression of MS. However, there is a lack of lipidomics studies in MS on CNS tissues, such as normal-appearing white matter (NAWM), where local inflammation initially occurs. Herein, we developed an untargeted reverse phase ultra-performance liquid chromatography time of flight tandem mass spectrometry (RP-UPLC-TOF MS^E^)-based workflow, in combination with multivariate and univariate statistical analysis, to assess significant differences in lipid profiles in brain NAWM from post-mortem cases of PPMS, SPMS and controls. Groups of eight control, nine PPMS and seven SPMS NAWM samples were used. Correlation analysis of the identified lipids by RP-UPLC-TOF MS^E^ was undertaken to remove those lipids that correlated with age, gender and post-mortem interval as confounding factors. We demonstrate that there is a significantly altered lipid profile of control cases compared with MS cases and that progressive disease, PPMS and SPMS, can be differentiated on the basis of the lipidome of NAWM with good sensitivity, specificity and prediction accuracy based on receiver operating characteristic (ROC) curve analysis. Metabolic pathway analysis revealed that the most altered lipid pathways between PPMS and SPMS were glycerophospholipid metabolism, glycerophosphatidyl inositol (GPI) anchor synthesis and linoleic acid metabolism. Further understanding of the impact of these lipid alterations described herein associated with progression will provide an increased understanding of the mechanisms underpinning progression and highlight possible new therapeutic targets.

## 1. Introduction

Multiple sclerosis (MS) is an autoimmune, inflammatory neurodegenerative condition, which affects more than 400,000 people living in the USA and approximately 2.5 million people worldwide [1]. The majority (85%) of MS patients are initially diagnosed in their third decade of life, with a relapsing and remitting clinical course. In most of these patients, after 10–15 years of disease duration, relapsing/remitting MS (RRMS) develops into secondary progressive MS (SPMS) [2,3]. Approximately 15% of MS patients however develop a progressive course from the onset, without preceding periods of relapse and remission, known as primary progressive MS, (PPMS), with a peak onset in the fifth decade of life, which is similar to the age when there is a conversion of patients with RRMS into SPMS [4,5]. It is currently not known whether PPMS and SPMS are distinct entities or whether they could be phenotypic variations of the same disease [6]. Whilst the inflammatory component of MS, which underpins relapses, is relatively well understood, the progressive neurodegenerative phase of the disease, in both PPMS and SPMS, is yet to be fully elucidated at the molecular level. Diffuse changes in normal-appearing white matter (NAWM) in the central nervous system (CNS) reported in progressive disease, include axonal damage, microglial activation and changes at the Node of Ranvier [7,8]. The neuronal/axonal damage and loss has been reported to underpin the progression of disability in PPMS [9]. Understanding the underlying pathogenesis of clinical progression in MS at the molecular and cellular level is vital for the development of therapies targeting the neurodegenerative process.

Lipid metabolism has been suggested, previously to play a significant role in the pathophysiology of MS [10,11]. Lipids involved in myelin formation and ensheathment of axons have been of most interest to researchers investigating MS pathogenesis, considering the hallmark demyelinated plaques or lesions in the CNS and the limited remyelination that takes place [8]. However, in addition to myelination, lipids in the CNS have a diverse range of functions, including cell signalling, exosome formation and cell-cell communication [12,13]. Laule et al. [14] described diffuse changes in the white matter (WM) in MS were a result of a primary lipid abnormality. Subtle changes in the WM lipid components, identified by our recent Raman spectroscopy data [15] highlight that apart from possibly impacting on the molecular packing of myelin membranes, these changes may impact on critical cellular processes involving lipids, which are vital for maintaining neural integrity and cell communication in the CNS.

There is an emerging body of previous metabolomic [16,17,18,19,20,21,22,23] and lipidomic [24,25,26,27,28,29,30,31,32,33,34] studies in MS and its various forms (i.e., RRMS, PPMS, SPMS) and these mainly involve biofluids, such as plasma, serum, cerebrospinal fluid (CSF), tears and urine, reviewed here [35,36]. Liquid chromatography mass spectrometry (LC-MS) has been previously applied to the analysis of CNS lipids. However, there is a lack of lipidomics reports on brain tissues such as NAWM, where the onset of MS disease occurs, and more particularly studies that differentiate PPMS from SPMS based on their lipidomic profile. Trepanier et al. [33] evaluated the concentration of oleic acid following demyelination and remyelination in the cuprizone mouse model of MS, to test if these changes occurred in specific lipid species. The group measured concentrations of fatty acids in total brain lipids and a panel of lipid species with phosphatidylcholine (PC). Compared to control, oleic acid was decreased after five weeks of cuprizone treatment and increased during the recovery phase. This decrease in oleic acid was associated with a specific decrease in the PC (36: 1) pool. Wheeler et al. [37] showed that despite no overall loss of lipid mass in MS NAWM and normal-appearing grey matter (NAGM) tissue in human MS and control post-mortem cases, a reduction in sphingolipids (SLs) and a gain in phosphatidylethanolamine (PE) species was observed using an electrospray ionization tandem mass spectrometry (ESI-MS/MS) shotgun approach. Among lipids, increased CSF or blood levels of cholesterol, which is an essential component of cell membranes and myelin, and its turnover products, was reported to be associated with adverse outcomes in MS patients, based on a review of 21 studies [38]. Whereas omega-3 fatty acid, alpha-linolenic acid, was protective when fed to mice with experimental autoimmune encephalomyelitis (EAE) and this was through preservation of the blood brain barrier [39]. Thus, evidence from the literature indicates that alterations in the content and levels of different lipid classes can influence disease progression in MS.

Herein, an untargeted reverse phase ultra-performance liquid chromatography time of flight tandem mass spectrometry (RP-UPLC-TOF MS^E^)-based workflow, in conjunction with multivariate (MVA) and univariate analysis, was developed, which can identify lipid alterations with high sensitivity and specificity in WM from control and NAWM from PPMS and SPMS post-mortem cases. We demonstrate that we can separate control cases from MS cases and that progressive disease, PPMS and SPMS, can be differentiated on the basis of the lipid profile of NAWM with good sensitivity, specificity and prediction accuracy based on receiver operating characteristic (ROC) curve analysis. Pathway analysis was also carried out revealing the most highly altered lipid pathways between PPMS and SPMS were glycerophospholipid metabolism, glycerophosphatidyl inositol (GPI) anchor synthesis and linoleic acid metabolism.

## 2. Results

### 2.1. Post-Mortem Case Details

Initially 12 blocks of tissue from each group, WM from controls and NAWM from SPMS and PPMS, were screened by H&E, Oil red O and Luxol fast blue histological stains to ensure that blocks with only WM or NAWM were selected. These were then further characterized using IHC to assess for loss of MOG and expression of HLA DR to ensure the samples selected were histologically comparable. Thus, following this screening process, white matter from nine control, and NAWM from eight PPMS and seven SPMS cases were analysed. The clinical and demographic parameters of these cases are listed in Table 1. All parameters (sex, age, PMI) did not differ significantly between pair-wise group comparisons (i.e., PPMS vs. SPMS, controls vs. PPMS, controls vs. SPMS) after one-way ANOVA test was performed. For sex, non-parametric test was performed (Kruskal–Wallis with post-hoc Tukey test) whereas for age and PMI parametric test (ordinary one-way ANOVA with post-hoc Tukey test) was applied.

### 2.2. Immunohistochemistry of White Matter Tissue Sections

Microscopic assessment of all tissue blocks following IHC, demonstrated no change in staining for myelin oligodendrocyte glycoprotein (MOG), which is expressed by oligodendrocytes, the myelin-producing cells in the central nervous system and representative of intact myelin. Microglia density and morphology, which was assessed by staining for the human leukocyte antigen (HLA DR) were comparable between the three groups, indicative of a low level of inflammation, as HLA DR plays a role in antigen presentation as part of the autoimmune response in MS. The IHC images from a control case and an MS case for MOG and HLA-DR staining are provided in Supplementary Figure S1 (Supplementary Materials). The images illustrate that there were no differences in staining between the control normal cases and the MS case, which provided the basis for classification of samples as normal-appearing white matter.

### 2.3. Multivariate Analysis of the RP-UPLC-TOF MS^E^ Data

**Figure 1:**
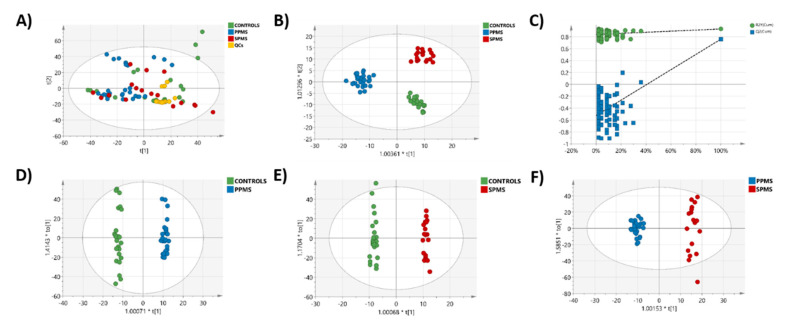
Lipidomic profile by RP-UPLC−TOF-MS^E^ in positive-ion mode. (**A**) PCA model between control (green), PPMS (blue) and SPMS (red) groups. QCs (yellow) are included. (**B**) OPLS-DA between the three groups (controls, PPMS, and SPMS). (**C**) A permutation test performed with 100 random permutations on the generated OPLS-DA model showed no overfitting of the model (Q2 = (0.0, −0.546)). Cross-validated OPLS-DA models between (**D**) controls and PPMS (R2X (cum) = 0.675, R2Y (cum) = 0.993, Q2 (cum) = 0.839), A = 1 + 6 + 0, CV-ANOVA: *p* = 2.73901e^−10^. (**E**) Controls and SPMS (R2X (cum) = 0.672, R2Y (cum) = 0.994, Q2 (cum) = 0.818). A = 1 + 6 + 0, CV-ANOVA: *p* = 1.07561e^−6^ and (**F**) PPMS and SPMS groups (R2X (cum) = 0.627, R2Y (cum) = 0.985, Q2 (cum) = 0.682). A = 1 + 5 + 0, CV-ANOVA: *p* = 3.74359e^−5^.

**Figure 2:**
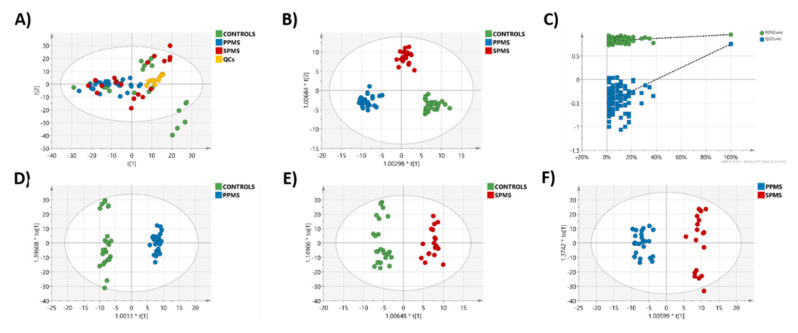
Lipidomic profile by RP-UPLC−TOF-MS^E^ in negative-ion mode. (**A**) PCA model between control (green), PPMS (blue), and SPMS (red) groups. QCs (yellow) are included. (**B**) OPLS-DA between the three groups (controls, PPMS, and SPMS). (**C**) A permutation test performed with 100 random permutations on the generated OPLS-DA model showed no overfitting of the model (Q2 = (0.0, −0.53)). Cross-validated OPLS-DA models between (**D**) controls and PPMS (R2X (cum) = 0.607, R2Y (cum) = 0.987, Q2 (cum) = 0.828) A = 1 + 5 + 0, CV-ANOVA: *p* = 5.23952e^−10^. (**E**) Controls and SPMS (R2X (cum) = 0.593, R2Y (cum) = 0.96, Q2 (cum) = 0.808) A = 1 + 4 + 0, CV-ANOVA: *p* = 1.52438e^−8^ and (**F**) PPMS and SPMS groups (R2X (cum) = 0.474, R2Y (cum) = 0.961, Q2 (cum) = 0.704) and A = 1 + 3 + 0, CV-ANOVA: *p* = 7.52298e^−7^.

The QC samples were tightly clustered in the PCA scatter plot in both positive and negative ion-mode (Figure 1A and Figure 2A) showing that our lipidomics RP-UPLC-MS^E^ method was valid, producing high quality data for further downstream statistical analysis. The three groups were well separated in the cross-validated OPLS-DA models (Figure 1B and Figure 2B). Additionally, when pair-wise comparisons were performed, all OPLS-DA models showed good separation between the two groups (Figure 1D–F and Figure 2D–F). The OPLS-DA permutation plot had low (negative) value for Q2-intercept, confirming validation of the original model between the three groups (Figure 1C and Figure 2C). Permutation plots for all pairwise OPLS-DA models are shown in Figure S2A–C (Supplementary Materials) (positive ion mode) and Figure S3A–C (Supplementary Materials) (negative ion mode).

**Figure 3:**
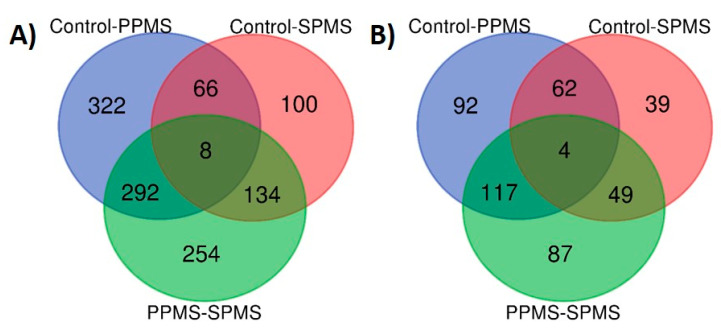
Venn diagrams of significantly altered lipids identified from pair-wise comparisons between groups, indicating the numbers of common and unique species in (**A**) positive and (**B**) negative ion mode. The raw abundances of ions/features are provided in supplementary data excel files Table S2a,b (Supplementary Materials).

Ions (features) derived from the OPLS-DA models were considered statistically significant when they exhibited a VIP value of >1 and a *p* < 0.05 (Student’s *t*-test, unequal variance) in group pair-wise comparisons. The raw data of the ions/features presented in Figure 3 are provided in the supplementary data files (Excel files, Table S2a,b (Supplementary Materials)). From the Venn diagrams, the unique ions/features that account for the separation of PPMS versus SPMS groups in positive and negative mode (Figure 3A,B) were selected.

From 254 (positive ion mode) and 87 (negative ion mode) features, only lipids that were identified with LIPIBLAST online library based on their MS^E^ spectra were considered for further downstream statistical analysis. Next, lipids that had an HMDB or a LIPIDMAPS ID number were chosen. A total of 76 lipids that were identified by MS^E^ spectra and had an HMDB or LIPIDMAPS ID number were found. Then, correlation analysis (Pearson’s or Spearman’s), depending on the distribution of the data being parametric (Pearson’s) or non-parametric (Spearman’s), was performed between the levels of lipids and clinical/demographic parameters i.e., age and post-mortem interval (PMI). In addition, a parametric Welch’s *t*-test or the non-parametric Mann–Whitney U-test was performed between the levels of lipids and the sex parameter. Lipids that were statistically correlated with these parameters were excluded from further downstream analysis (see supplementary data Table S3 (Supplementary Materials)). Therefore, the 44 lipids that survived the sequential correlation analyses were considered as statistically significant to differentiate PPMS and SPMS. These lipids are presented in Table 2, for both positive and negative ion mode.

Furthermore, ions that were common in separating controls from PPMS and controls from SPMS, as depicted in Venn diagrams (Figure 3A,B), for positive and negative ion mode, respectively, were also selected as markers of controls against the two forms of progressive MS disease (i.e., PPMS and SPMS). After excluding lipids where levels showed a significant association with sex, age or PMI, 10 lipids were found to be significant in differentiating controls from PPMS and SPMS combined. These 10 lipids are shown in Table 3.

### 2.4. Markers of Progression between PPMS and SPMS

After following all the steps in the lipidomics workflow described in 3.2, 44 lipids were identified as significant markers of progression in PPMS compared to SPMS (Table 2). Overall, most of these lipid levels (29/44 or 65.9%) were decreased in SPMS compared to PPMS, while 15/44 (or 34.1%) were increased in SPMS compared to PPMS. Additionally, when lipids were sorted by class, mixed mode effects were observed. All 9 phosphatidylethanolamines (PEs) were decreased in SPMS compared to PPMS, as well as the majority (5/7) of putative PE-plasmalogens (PE-P). These are putative assignments as the location of the double bonds with respect to the ether linkage cannot be assigned. Future experiments using specially designed LC-MS methods can confirm these assignments. Regarding other phospholipids (PLs), i.e., phosphatidylanisols (PA), phosphatidylinositols (PI), phosphatidylserines (PS), again the same trend of lower levels in SPMS was observed for the majority of lipids; 3/5 PA, 7/9 PS, and 2/2 PI had decreased levels in SPMS. On the contrary, amongst the lipids that exhibited higher levels in SPMS (compared to PPMS) were 1 ceramide (Cer), 3 diacylglycerols (DG), 2/3 phosphatidylglycerols (PG), 1/2 phosphatidylcholines (PCs), and 2/3 lysophosphatidyl lipids (lysoPLs). Notably, all 44 lipids exhibited peak areas with RSD < 30% in the QC samples; 28/44 (or 63.6%) lipids had RSD < 20% in the QCs, which shows that these 44 significantly altered lipids in SPMS versus PPMS are stable throughout the LC-MS analysis time, which spanned over four days.

**Figure 4:**
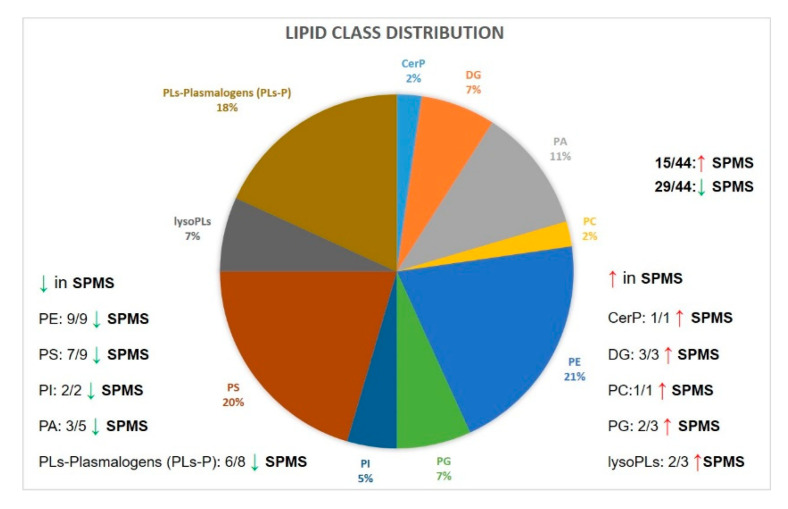
Pie chart showing the lipid class distribution of significantly altered lipids and the trend between PPMS and SPMS (positive and negative ion mode combined). The three most abundant lipid classes are PE, PS and PLs-P. PE, phosphatidylethanolamines; PS, phosphatidylserines; PLs-P, putative plasmalogens.

Furthermore, although the fold change (FC) between SPMS and PPMS was small for the majority of lipids, when the lipids were investigated together, they formed a “lipid signature” that could provide further information about the possible different mechanisms of disease progression between PPMS and SPMS. Notably, 4 lipids had a FC > 1.5 (lysoPC (10:0), PG (18:0_16:0), PS (18:1_20:3) and lysoPE (22:2)), in SPMS versus PPMS, with two of them being lysoPLs (lysoPC and lysoPE); this shows that lysoPLs are highly abundant in SPMS (compared to PPMS) to a much higher degree than the other significant lipids. Additionally, the three most abundant lipid classes are PE, PS and PLs-P. PE, phosphatidylethanolamines; PS, phosphatidylserines; PLs-P, putative plasmalogens as can be observed in the pie chart below (Figure 4).

### 2.5. Lipid Markers of Progression in MS Compared with Control Cases

Ten lipids were found to be common and statistically significant for the separation of controls and PPMS, and the separation of controls and SPMS, after applying the same lipidomics workflow: seven lipids in negative ion mode and three lipids in positive ion mode. Of all 10 lipids, most of them in both SPMS and PPMS NAWM, demonstrated the same trend when compared with control WM samples. As can be seen in Table 3, the levels of 8/10 lipids were lower in both PPMS and SPMS compared to controls, while 2/10 lipids were found to be increased in both PPMS and SPMS in relation to the control group. The two lipids that increased in PPMS and SPMS were identified as PE (18:2_20:0) and PE (20:4_20:0). PE (20:4_20:0) contains one fatty acid (FA) with a 20-carbon chain and four double bonds; this FA is assigned to eicosanoid arachidonic acid (AA) which is an omega-6 (n-6) polyunsaturated fatty acid (PUFA).

Furthermore, the 8 lipids that were attenuated in both PPMS and SPMS compared to controls, were identified as PA, PE, PI, PS, DG and sulfatide. Most of these lipids (five out of eight), contain in their structure the FAs 18:0, 18:1 and 18:2, which are the three most abundant FAs in human plasma [40]. Additionally, PE (22:6_22:0) and PA (20:5_18:1) contain the FAs 22:6 and 20:5, which are assigned to oxylipins (pro-resolvins) docosahexaenoic acid (DHA) and eicosapentaenoic acid (EPA). Lastly, our lipidomics workflow revealed the presence of odd chain saturated fatty acids (OCS-FAs) incorporated in phospholipids, such as PA (18:2_17:0) and PS (18:2_19:0).

### 2.6. ROC Analysis

**Figure 5:**
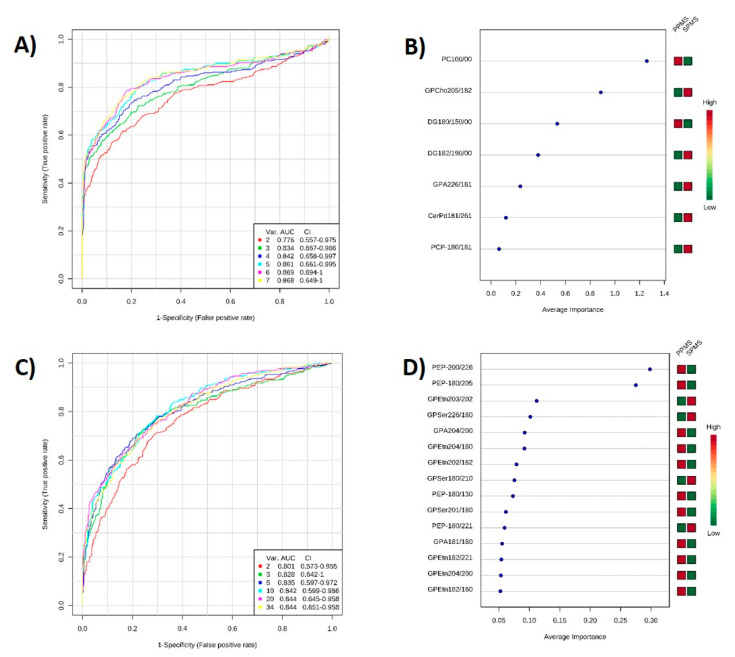
ROC validation analysis of lipid putative biomarkers between PPMS and SPMS in (**A**,**B**) positive and (**C**,**D**) negative ion mode. Comparison of different variables based on ROC curves: the legend shows the feature numbers and the AUCs of the six models for (**A**) positive, and (**C**) negative ion mode. The average importance of seven lipids in positive ion mode (**B**), and 34 lipids (top 15 shown) (**D**) in negative ion mode, based on ROC curves, in descending order of importance. The prediction of the model depends on the area under the curve (AUC) provided by ROC analysis: the greater the AUC, the better the prediction of the model.

ROC curve analysis was performed to validate the OPLS-DA analysis and test the applicability of statistically differential lipids in separating PPMS and SPMS. Figure 5A,C show a group of ROC curves for models established by using different lipids selected by the filter approach. Six models were generated for both positive (Figure 5A) and negative (Figure 5C) ion mode. For positive ion mode, the top two lipids lysoPC (10:0) and PC (20:5_18:2) were used to build classification model 1; the area under the curve (AUC) value was 0.776 and the 95% confidence interval (CI) was 0.557–0.975. When all seven lipids were used, the AUC value was 0.868, while sensitivity was 77.8%, specificity of 92.6% and predictive accuracy of 81%. For negative ion mode, when all 34 lipids were used the AUC value was 0.844, sensitivity was 66.7%, specificity of 83.3% and predictive accuracy of 74.4%. Based on the selected significant lipids, ROC curve analysis revealed that the two OPLS-DA models identified lipid markers of progression in NAWM, sorted by their importance (Figure 5B,D) and these account for the differences between PPMS and SPMS.

### 2.7. Metabolic Pathway Analysis

**Figure 6:**
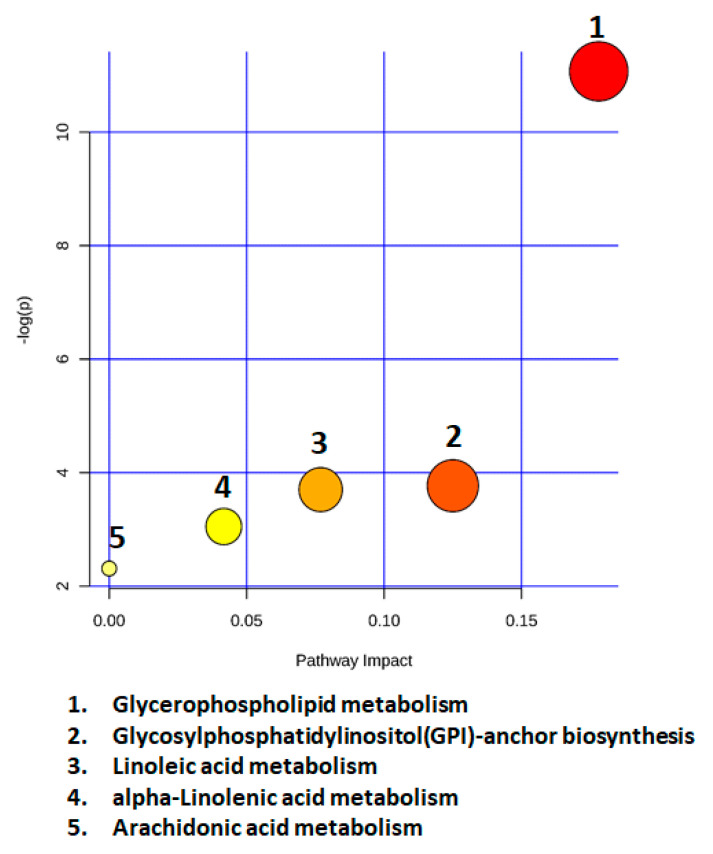
Metabolic pathway analysis of statistically significant lipids between PPMS and SPMS groups, combined in positive and negative ion mode. The pathways depicted are listed from 1 to 5 in descending order of importance, based on a combination of both the *p* values (*y*-axis) and impact (*x*-axis), according to Metabolic Pathway Analysis (MetPA) carried out in MetaboAnalyst 4.0.

Lipid species identified as potential markers (positive and negative ion mode combined) of progressive disease in NAWM were related to glycerophospholipid metabolism [PC, PE, LysoPC (18:1)], glycosylphosphatidylinositol(GPI)-anchor biosynthesis (PE), linoleic acid (PC), alpha-linolenic acid (PC) and arachidonic acid metabolism (PC) (Figure 6). The pathways were considered as the metabolic routes most significantly altered in PPMS compared to SPMS, based on a combination criteria of lowest *p* values (*p* < 0.05) in the *y*-axis and pathway impact in the *x*-axis. Glycerophospholipid metabolism was the most significantly altered pathway between PPMS and SPMS.

### 2.8. Correlation Analysis of Lipid Levels with Sex, Age, and PMI

As stated previously, several identified lipids which were found to be significantly altered between PPMS and SPMS, and for which an HMDB or LIPIDMAPS ID number was associated with their name, were correlated with sex, age, and PMI. In total, levels of 32 lipids were found to be correlated with at least one of these parameters. Of these 32 lipids, 17 lipids were correlated with age, 14 lipids correlated with PMI and two lipids with sex. More specifically, most lipids that were correlated with age showed a positive correlation (12/17 or 70.6%) while 5/17 (or 29.4%) showed a negative correlation. Of the 14 lipids statistically associated with PMI, 12/14 (or 85.7%) were negatively correlated with PMI, while two lipids showed a positive correlation. Lastly, of the two lipids that were associated with sex, PE (P-18:0_22:1) was statistically increased in females while PE (18:3_21:0) had significantly higher levels in males. A detailed description of these 33 lipids and their correlation with sex, age and PMI is given in Supplementary File, Table S3 (Supplementary Materials).

## 3. Discussion

Lipidomics is a powerful tool for the study of neurological diseases such as MS [41]. It has been used to determine subtle changes in the lipidome of biofluids and tissues from MS cases when compared with healthy control samples [24,27,29,34]. Recent lipidomic and metabolomics studies have also investigated different forms of MS, however there is a lack of lipidomic studies on differentiating PPMS from SPMS. Although different biofluids that could be used as biomarkers for MS were studied for their lipidomic profile, to the best of our knowledge there are no lipidomics reports on CNS tissue, including NAWM, where early molecular changes, prior to the onset of disease, could be detected. This becomes even more evident considering the wealth of information available from previous immunohistology reports [42,43]. More importantly, imaging mass spectrometry (IMS) techniques (i.e., MALDI-MS, LESA-MS, DESI-MS) have very recently proven to be effective in investigating the lipidome of various neurodegenerative diseases, such as Alzheimer’s, Parkinson’s, and MS, reviewed here [44]. Thus, spatiotemporal lipid changes in brain tissues can be mapped to monitor the progression of disease, as was recently described by Bergholt et al. [45] for MS. Therefore, lipid biomarkers from homogenates of NAWM, using a RP-UPLC-TOF MS^E^ method, can be compared and complement lipidomics data from IMS studies, providing a deeper insight into the role of lipids in progression of MS disease.

In this study, we developed an untargeted lipidomics workflow based on RP-UPLC-TOF MS^E^ method, combined with MVA and univariate statistical analysis, to demonstrate that NAWM from PPMS and SPMS post-mortem cases, can be differentiated based on their lipidomic profile, but also when compared with healthy control white matter post-mortem samples. After applying the lipidomics workflow developed here, the identified significantly altered lipids between PPMS and SPMS, and between both PPMS and SPMS compared to controls, belong to three key families: (a) sphingolipids, (b) phospholipids, and (c) glycerolipids. To the best of our knowledge this is the first report of altered lipid profiles in NAWM comparing PPMS and SPMS cases using lipidomic methods. Most recent studies focus on differentiating mainly RRMS and SPMS, using biofluids. Herein, we discuss the key differences and the role of these significantly altered lipid families in PPMS and SPMS NAWM and control WM.

Phospholipids are the main class of lipids shown in our study to differentiate between the two forms of MS progression. More specifically, 40 out of 44 significant lipids belong to this class, which comprises different subclasses: PC, PE, putative plasmalogens PC and PE (PC-P, PE-P), phosphatidic acids (PA), phosphatidylglycerol (PG), phosphatidylinositol (PI), phosphatidylserines (PS) and lysophospholipids (lysoPLs), lysoPE and lysoPC. Twenty-nine out of 44 phospholipids (66%) were decreased in SPMS compared to PPMS NAWM post-mortem brain samples, while 34% were increased in SPMS compared to PPMS.

Phospholipids are the most abundant lipid class present in human plasma [46]. Recent studies have demonstrated that phospholipids can discriminate between MS and healthy control CSF and plasma. The group of Stoessel in 2018 reported reduced levels of five PCs and four lysoPCs in plasma of PPMS patients compared to healthy controls, sex and age matched, using a metabolomic approach with LysoPC (20:0) being statistically decreased in the MS cohort over a 24-month period [23]. In addition, an untargeted LC-MS approach was performed by Nogueras et al. [27] to determine global lipidomic differences in the CSF between MS and non-MS patients, i.e., those who had had a lumbar puncture but the diagnosis was not MS. Forty seven lipid species were identified, among which there were 30 glycerolipids, five sterol lipids, four FAs and five phospholipids (PC, PE, and PS). Another study reported that alpha-linolenic acid administered to mice with EAE was protective against the disease through reducing the permeability of the blood brain barrier [47]. Furthermore, Pieragostino et al., in 2015, analysed the hydrophobic metabolites of MS and other neurological disease (OND) patients’ CSF using MALDI-TOF mass spectrometry [30]. Their studies demonstrated altered levels of specific phospholipids in the MS group compared to the OND group. They reported a significantly increased level of lysoPC (18:1), lysoPC (18:0), and lysoPI (16:0) in the CSF of MS patients. LysoPC levels correlated to the IgG Index, indicative of breakdown of the blood brain barrier. Additionally, the levels of lysoPI in MS were negatively correlated to the Expanded Disability Status Scale (EDSS) score suggesting that increased levels of lysoPI (16:0) in CSF could exhibit a protective role against development of neurological symptoms in MS patients. In the current study, analysis of NAWM from PPMS and SPMS samples found lysoPC (10:0) and lysoPE (22:2) were significantly increased in SPMS compared to PPMS (2- and 1.5-fold, respectively), whereas lysoPC (17:0) was decreased by 22.5%. Further analysis of the function of these lipids in the CNS is required to fully understand the implications of these changes in tissues.

Furthermore, PC is the major phospholipid species of eukaryotic membranes and removal of one of the fatty acids results in generation of lysoPC via the phospholipase A2 (PLA2) enzyme. It has been previously reported that PLA2 products are involved in various pathways, such as signal transduction, biosynthesis of inflammatory mediators, differentiation and apoptosis [48]. There is evidence that a deregulation of PLA2 and its products has been associated with various neurodegenerative diseases, such as Alzheimer’s disease, Parkinson’s disease, and amyotrophic lateral sclerosis [49]. The therapeutic effect of Fingolimod, a sphingosine-1-phosphate (S1P) analogue approved for treatment of MS patients, acts via inhibition of PLA2 activity in the CNS [50]. Therefore, it is suggested that the pathological overstimulation of PLA2 contributes to releasing lysoPCs from membrane phospholipids, resulting in accumulation of lysoPC species in brain tissue, confirmed by high levels of lysoPCs in CSF of MS patients [23]. Herein, we found that of the two lysoPCs identified as significantly different between PPMS and SPMS, one was upregulated and the other downregulated, which confirms their complex role in progression of MS.

The myelin sheath is extremely rich in sphingolipids and glycerophospholipids [51]. Approximately 70–85% of the myelin dry weight is lipids [52]. The major FAs observed in myelin are stearic acid [FA (18:0)] and palmitic acid (FA (16:0)), saturated fatty acids, as well as oleic acid (FA (18:1)), a monounsaturated fatty acid [53,54]. Previous studies have shown a decrease in oleic acid (FA (18:1)) in the WM of post-mortem brain of MS cases compared to healthy controls [55,56]. FAs are the building blocks that incorporate into the glycerol backbone of membrane phospholipids, comprising the sn-1 and sn-2 side chains. It was observed in the current study that 11/44 (25%) of significantly altered lipids (PPMS vs. SPMS) have oleic acid (FA (18:1)) side chains. Seven out of 11 (63.6%) oleic acid moieties incorporated in phospholipids are decreased in SPMS vs. PPMS; this agrees with these previous reports, when WM from MS cases were compared with control cases [55,56]. Although we did not perform a targeted FA profile here (commonly performed by GC-MS), thereby the exact FA structure could only be tentatively identified, the fact that FA (18:1) is one of the most highly abundant FA in human myelin would support its identification here.

Glycerolipids also showed differences in lipid profiles between PPMS and SPMS cases. Specifically, an upregulation of diacylglycerols (DG) in SPMS vs. PPMS was determined. This finding could be linked with a defect of the diacylglycerol acyltransferase (DGAT) enzyme, which, in turn, is related to insulin resistance, an observation recently seen in patients with MS [57,58]. Also, a recent study revealed that inhibition of DGAT enzyme blocked the accumulation of lipid droplets consisting of neutral and phospholipids measured by LC-MS in cell cultures of murine cortical astrocytes [59]. DGAT catalyses the final acylation step in the triaclyglycerol (TG) biosynthetic pathway by transferring a fatty acyl group from acyl-CoA to the sn-3 position of diacylglycerol to form TG. Although this study compared controls versus MS patients and not SPMS vs. PPMS, it is indicative of the role of DGAT enzyme in MS and subsequently an indirect role for DG in MS.

Sphingolipids were the third class of lipids that was found to differentiate between PPMS and SPMS. Sphingolipids are integral components of biomembranes, which are involved in many cellular functions, including cell proliferation, signalling cascades and apoptosis [60]. Sphingolipids include sphingomyelins (SMs), ceramides and sphingosines, involved in the same pathway, by sequential enzymatic reactions [48]. It is noteworthy that fingolimod, which as mentioned beforehand here, is an antagonist of sphingosine-1-phosphate and is an effective treatment for MS [61]. This highlights the importance of this lipid family in MS, levels of which we have demonstrated to be altered in NAWM between PPMS and SPMS. Moreover, patients with MS have a higher level of ceramide C16:0 and C24:0 in the CSF, which may be linked to the axonal damage observed in the disease [62]. Ceramide is produced in the CSF through the hydrolysis of sphingomyelin. In addition, lower sphingolipid content was found in NAWM from MS cases compared to controls [37], while sphingosine content is increased in MS NAWM [63]. Although there are no previous reports on differences in ceramide levels between SPMS and PPMS patients in NAWM, the abovementioned studies clearly demonstrate the key role of ceramides in MS.

Hence, our results agree with other studies that have found alterations in lipids, such as ceramides in CSF [64], brain PCs [33] and sphingolipids in the WM of the brain [37]. Finally, NAWM in human brain tissue [33] is composed mainly of PCs and PEs, levels of which differed between PPMS and SPMS groups in our study. Overall, lipid level alterations in NAWM could reflect alterations in the lipid composition of the myelin sheath as well as cell membranes of neurons and glia, revealing underlying mechanisms in the progression in MS. One limitation of this study could be the number of cases examined and that samples were post-mortem brain tissues, although any lipid changes linked to post-mortem interval (PMI) were removed from the study. Hence, lipid markers of MS progression identified here cannot be used for biofluid biomarkers between PPMS and SPMS, although these should be further investigated in for example CSF to assess their potential as biomarkers of disease. However, we demonstrate herein that our approach could distinguish PPMS from SPMS cases, but also PPMS and SPMS from controls, based on their lipidomic signature in CNS tissue; these findings could complement and add more validation to lipid biomarkers proposed in other lipidomics studies of biofluids.

A group of 10 lipid markers were identified to separate controls from progressive forms of MS (PPMS and SPMS), whereas 44 lipids were significantly altered between PPMS and SPMS. Different panels of significant lipids were hence found to account for these separations; however, it should be noted that phospholipids were the dominant differentiating lipid species for both comparisons. Lastly, odd chain saturated fatty acids (OCS-FAs) incorporated in phospholipids, such as PA (18:2_17:0) and PS (18:2_19:0), were among the significant lipids differentiating controls from PPMS and SPMS. Although these lipids were considered of non-mammalian origin, there is emerging evidence that increased consumption of dairy products has an association with an increase in blood plasma OCS-FAs [64,65].

Therefore, although the scope of our study focused on finding markers to differentiate PPMS and SPMS, our data favour that semi-targeted lipidomics UPLC-MS/MS approaches (i.e., phospholipid profiling) would be beneficial in studying progressive MS. These approaches would aid in a more comprehensive discovery of both diagnostic and prognostic markers of MS disease, ultimately aiding to unravel the complex mechanisms that govern progressive MS disease.

## 4. Materials and Methods

### 4.1. Post-Mortem CNS Cases

The human brain tissue used in this study was provided by the UK MS Society Tissue Bank, Imperial College, London, UK. Ethical approval for the collection of the post-mortem tissue samples was approved by Wales Research Ethics Committee (reference number 08/MRE09/31) and Sheffield Hallam University, Faculty of Health and Wellbeing Research Ethics Committee approved the study. The summarized characteristics of the cases used in this study are detailed in Table 1, with information for each case provided in supplementary data (Table S1 (Supplementary Materials)).

### 4.2. Acquisition of Lipidomic Data

#### 4.2.1. Analytical Standards

LC/MS grade Acetonitrile (ACN) and isopropanol (IPA) was obtained from Fisher Scientific (Loughborough, UK). Distilled Water (18.2 MW) for chromatographic separation was purified in a Milli-Q device (Millipore, Merch Darmstadt, Germany). Ammonium formate (NH_4_HCO_2_) and formic acid were purchased from Sigma Aldrich (Gillingham, UK). PBS solution and bovine serum albumen (BSA) powder were purchased from Fisher Scientific (Loughborough, UK). Monoclonal antibody (anti-HLA-DR) was purchased from Abcam (Cambridge, UK) while the avidin biotin peroxidase complex ABC kit and peroxidase substrate were obtained from Vector Laboratories (Peterborough, UK).

#### 4.2.2. CNS Sample Collection

White matter (WM) from control (CON) cases (n = 8) and NAWM from MS post-mortem cases, PPMS (n = 9) and SPMS (n = 7) were included in the study. Confirmation of the classification of MS cases as primary or secondary progressive was obtained by the MS Society Tissue Bank neurologists’ assessment of the patient clinical notes pre-mortem. Serial 30 µm sections were acquired in a cryostat (Leica, UK) and were transferred into cold (−20 °C) pre-labelled and weighed Eppendorf tubes. Tissue weights collected were in the range of 10–20 mg and these were stored at −80 °C, until used for lipid extraction. For each brain block sample, three biological replicates were acquired. Serial sections (10 µm) from each block were collected on polysine coated glass slides (VWR, Lutterworth, UK) and stored in airtight containers at −80 °C until required for histology and immunohistochemistry (IHC).

#### 4.2.3. Classification of WM and NAWM Tissue Using Histological and Immunohistochemical Techniques

Serial frozen sections (10 µm) were used to (a) assess that only WM was acquired for analysis, based on Hematoxylin and Eosin (H&E) and Oil Red O and Luxol Fast Blue staining to ensure no evidence of demyelination and (b) confirmation of this by IHC was performed with a monoclonal antibody to myelin oligodendrocyte glycoprotein (MOG) (provided by Professor C. Linington, Glasgow University) and to assess the extent of any inflammation, evidenced by activation of microglia, using increased expression of human leukocyte antigen (HLA-DR) with a monoclonal antibody, Abcam (Cambridge, UK), and to identify any change in morphology of resident microglia. Negative controls were obtained by replacement of the primary antibody with PBS with 0.05% bovine serum albumen (BSA). The avidin biotin peroxidase (ABC, Vector Laboratories, Peterborough, UK) method was used with diaminobenzidine as substrate, producing a brown positive stain, and hematoxylin counterstain, providing a blue nuclear stain as described previously [66]. Slides were assessed by two independent observers. Where there was any discrepancy between the observers, the sections were observed jointly to reach an agreed decision of the classification of normal and NAWM. Any tissue blocks that did not meet this classification were excluded from downstream analysis.

#### 4.2.4. Lipid Extraction from White Matter

Lipid extraction was performed as described in a recent publication [67], with minor modifications as follows. Brain tissues in Eppendorf tubes were briefly centrifuged for 2 min at 4000× *g*, at 4 °C. Methanol was added to each tube and the volume adjusted for each sample weight to ensure a consistent volume/weight ratio. Tissues were vortex-mixed for 40 s, followed by disruption and homogenization in a TissueLyser LT (Qiagen, Manchester, UK) for 2 min at 30 Hz at room temperature. A volume of 200 µL of homogenate was recovered from each tube and transferred into clean Eppendorf tubes. Then, 400 µL of chloroform was added and the sample vortexed for 30 s, followed by addition of 150 µL of water and vortex for 30 s. After a final centrifugation step at 4000× *g* for 10 min at 4 °C, the lower organic phase containing the extracted lipids was recovered and transferred into clean Eppendorf tubes. The samples were then dried using a vacuum concentrator 5301 (Eppendorf, Hamburg, Germany) for 60 min at 35 °C. Dried extracts were reconstituted in isopropanol (IPA)/acetonitrile (ACN)/water (4:3:1 *v*/*v*/*v*, 100 µL). A quality control (QC) sample was prepared by pooling aliquots (25 µL) of each processed tissue, 24 in total. Samples were transferred to brown glass autosampler vials and stored at −80 °C until LC-MS analysis.

#### 4.2.5. UPLC–MS Data Acquisition

A detailed protocol of the RP-UPLC–TOF MS^E^ parameters and instrumentation was recently described and adopted [68]. Briefly, a 20 min gradient mobile phase system was employed using a UPLC binary solvent manager. The mobile phase A consisted of ACN/water (60:40), 10 mM NH_4_HCO_2_, and 0.1% formic acid and mobile phase B of IPA/ACN (90:10), 10 mM NH_4_HCO_2_ and 0.1% formic acid. The flow rate was set at 0.4 ml/min, the injection volume to 3 µL and the autosampler temperature to 4 °C. The gradient profile was set as follows: 60–57% A (0.0–2.0 min), 57–50% A (2.0–2.1 min; curve 1), 50–46% A (2.1–12.0 min), 46–30% A (12.0–12.1 min; curve 1), 30–1% A (12.1–18 min), 1–60% A (18.0–18.1 min) and 60% A (18.1–20.0 min). An Acquity UPLC CSH C18, analytical column (2.1 × 100 mm, 1.7 μm) (Waters Corp, Milford, MA, USA) was used for chromatographic separation and maintained at 55 °C. Mass spectrometry was performed on a Waters Xevo G2-XS QTof (Waters Corp, Milford, MA, USA) equipped with an electrospray ionization (ESI) source in positive- and negative-ion mode. The electrospray voltage was 3 kV (−3 kV for negative), the cone voltage was 30 V, the source temperature was 120 °C and the desolvation temperature was set to 450 °C. Nitrogen was used as both desolvation gas (flow rate, 580 L/h) and the cone gas (50 L/h). Two parallel alternating scans were employed for low collision energy and high collision energy acquisition. Scan for low collision energy was set to 50–1200 *m/z*. The high collision energy scan was performed without ion selection (MS^E^ channel) [69]. The QC samples were run four times before the test sample sequence to stabilize the LC-MS system, and subsequently after every fifteen sample injections.

### 4.3. Data Processing and Lipid Identification

Acquired UPLC-MS data were processed using Progenesis QI (v.2.2) software (Nonlinear Dynamics, Newcastle upon Tyne, UK), for peak detection, alignment, deconvolution, ion filtering, compound identification and normalization using total area of features identified by the software. Ion filtering based on QCs was used. More specifically, an ion had to meet the following criteria to be selected for further downstream statistical analysis: (a) to have a CV <30% of peak areas in QCs, and (b) to follow the 80% rule, i.e., an ion had to have at least non-zero intensities in >80% for each group (controls, PPMS and SPMS samples) [70,71,72]. The retention times (RTs), *m/z* values and corresponding peak intensities were imported into SIMCA-15 (Sartorius Stedim Data Analytics AB, Umeå, Sweden) for MVA. Principal component analysis (PCA) and orthogonal partial least squares−discriminant analysis (OPLS-DA) were performed to investigate differences in lipidomic profile among the groups [73,74]. The variables of importance in the project (VIP) values [75] followed by two-tailed Student’s *t*-test (unequal variance) were used to identify significant lipids for pair-wise group comparisons (i.e., controls vs. PPMS, controls vs. SPMS, and PPMS vs. SPMS), adopting the protocol described in [76]. A *p*-value < 0.05, without corrections for multiple comparisons, between means of each pair-wise group comparison for each ion was used in order to detect as many lipids as possible, that would be meaningful if there were different clusters from separate classes found as potential biomarkers, as was the case. In addition, having three groups to compare, as opposed to the more standard two-group comparisons in a control vs. disease case, decreases the number of potential unique biomarkers, in the comparison between PPMS vs. SPMS disease. Notably, we adopted this strategy from a recent publication using metabolomics to identify biomarkers in drug induced liver injury, where the group also had three human patient groups to compare see reference [76].

**Figure 7:**
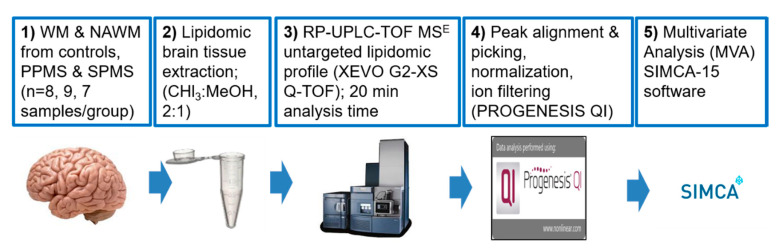
Lipidomics RP-UPLC-TOF MS^E^ based workflow.

The lipidomics workflow is depicted in Figure 7. For lipid structure assignment, accurate *m/z* measurements were matched to the accurate mass of lipids from online metabolomics databases, i.e., METLIN [77], HMDB v.4 [78], and LIPID MAPS [79]. After an assessment of retention time, experimental tandem MS spectra (MS^E^) were compared against MS/MS fragmentation patterns available in LIPIDBLAST [80,81] library embedded in PROGENESIS QI (v.2.2) (Nonlinear Dynamics, Newcastle upon Tyne, UK), software. Future work should validate these assignments using the MS/MS raw data.

ROC curve validation of significant lipids in positive and negative MS mode was performed in MetaboAnalyst [82] (http://metaboanalyst.ca). Metabolic pathway analysis (MetPA) was also performed in MetaboAnalyst to highlight lipid pathways significantly altered in SPMS and PPMS, and in controls versus PPMS and SPMS combined. Venn diagrams were generated to select the lipids separating groups, when pair-wise comparisons were made (i.e., PPMS from SPMS, but also controls versus PPMS and SPMS combined (http://bioinformatics.psb.ugent.be/webtools/Venn/).

## 5. Conclusions

In conclusion, our data, obtained by an RP-UPLC-TOF-MS^E^ lipidomic approach, suggest an altered lipid profile in NAWM from PPMS, SPMS compared with control samples. Lipidomic analysis led to the identification of 44 lipids which differentiated PPMS from SPMS. These lipids belong to classes of phospholipids, sphingolipids and glycerolipids, and were related to different biochemical pathways, mainly glycerophospholipid, GPI-anchor biosynthesis, linoleic acid and alpha-linolenic acid metabolism, consistent with previous studies. Since the NAWM tissue used in this study was selected based on the absence of inflammation and demyelination, using immunohistochemistry, indicates that the statistical differences observed in the lipidome by RP-UPLC-TOF MS^E^ were attributed to the progression of MS disease and not due to changes in the inflammatory status between PPMS and SPMS or related to ongoing demyelination. Although the findings need to be confirmed in a larger number of post-mortem cases, specifically using an independent second set of samples to externally validate the ROC curve analysis, the identification of these 44 lipids could provide valuable insight into the pathophysiology of progression in MS and lead to development of targeted therapies for progressive MS in the future.

## Figures and Tables

**Table 1:** Demographic and clinical parameters of cases ^a^.

	Healthy Controls (*n* = 8)	PPMS (*n* = 9)	SPMS (*n* = 7)	*p*-Value
Age (years)	76 ± 9	61 ± 10	60 ± 15	n.s.
Range	(63–88)	(39–73)	(39–88)	
Female/Male	2/6	6/3	6/1	n.s.
PMI (hours)	22.3 ± 8.5	14.6 ± 5.1	13.7 ± 6.3	n.s.
	(5–33)	(8–24)	(7–24)	

^a^ Data are mean ± SD, (range). *p*-values for comparisons between the PPMS and SPMS groups. A one-way ANOVA non-parametric (Kruskal–Wallis with post-hoc Tukey test) (sex) or parametric (ordinary one-way ANOVA with post-hoc Tukey test) (age, PMI) test was used for calculating *p*-values. Abbreviations: PMI, post-mortem interval; PPMS, primary progressive multiple sclerosis; SPMS, secondary progressive multiple sclerosis; n.s., non-significant (*p* > 0.05).

**Table 2:** List of significant lipids identified in normal-appearing white matter (NAWM) that differentiate PPMS and SPMS groups by RP-UPLC-TOF MS^E *a*^. Lipids are sorted in classes and listed alphabetically (ascending).

Peak Number	MS Mode	RT	*m/z*	Adduct	Lipid Identified	Delta (ppm)	*p*-Value	FC	Trend (SPMS vs. PPMS)	RSD(%) in QCs
1	pos	14.38	778.6076	M + Na	CerP (d18: 1/26: 1)	−1.123	0.04439	1.210	↑	21.65
2	pos	6.93	621.4856	M + K	DG (18: 0_15: 0)	0.176	0.00954	1.338	↑	11.19
3	pos	14.8	635.5615	M + H	DG (18: 2_19: 0)	0.970	0.02617	1.387	↑	27.39
4	pos	0.61	429.2722	M + NH_4_	lysoPC (10: 0)	−0.506	0.01166	2.144	↑	27.30
5	pos	1.84	527.3813	M + NH_4_	lysoPC (17: 0)	−1.301	0.03450	0.775	↓	18.24
6	neg	6.21	514.3291	M − H_2_O − H	lysoPE (22: 2)	−2.182	0.01040	1.521	↑	24.22
7	neg	13.73	683.5004	M − H_2_O − H	PA (18: 1_18: 0)	−2.372	0.02982	1.182	↑	17.39
8	neg	10.15	779.5376	M + Cl	PA (18: 1_21: 0)	1.749	0.00914	0.750	↓	27.26
9	neg	13.73	733.5153	M − H_2_O − H	PA (20: 4_20: 0)	−3.302	0.02392	1.418	↑	27.47
10	neg	6.77	885.6538	M + FA − H	PA (22: 2_24: 0)	−6.192	0.02793	0.709	↓	17.69
11	pos	9.79	746.4815n	M + H, M + NH_4_	PA (22: 6_18: 1)	−9.618	0.00450	0.762	↓	18.72
12	pos	8.43	821.5770	M + NH_4_	PC (20: 5_18: 2)	−4.189	0.03984	1.497	↑	19.01
13	pos	13.66	794.6051	M + Na	PC (P-18: 0_18: 1)	2.244	0.01342	0.747	↓	12.38
14	neg	13.66	780.593	M − H_2_O − H	PE (18: 1_22: 1)	2.113	0.02526	0.745	↓	25.62
15	neg	7.64	696.5034	M − H_2_O − H	PE (18: 2_16: 0)	8.412	0.00112	0.587	↓	21.90
16	neg	11.22	820.5576	M + Cl	PE (18: 2_21: 0)	−6.698	0.01061	0.796	↓	13.39
17	neg	8.92	832.564	M + Cl	PE (18: 2_22: 1)	1.436	0.01213	0.793	↓	26.52
18	neg	13.51	766.5446	M − H	PE (20: 2_18: 2)	7.043	0.00175	0.702	↓	17.09
19	neg	13.57	792.5594	M − H	PE (20: 3_20: 2)	5.652	0.01380	0.756	↓	27.45
20	neg	13.93	806.6094	M − H_2_O − H	PE (20: 3_22: 0)	3.015	0.01864	0.745	↓	18.16
21	neg	8.49	812.5500	M + FA − H	PE (20: 4_18: 0)	6.873	0.00507	0.515	↓	18.51
22	neg	10.28	840.5818	M + FA − H	PE (20: 4_20: 0)	7.300	0.02022	0.660	↓	11.71
23	neg	7.71	702.5488	M − H	PE (P-16: 0_18: 0)	6.340	0.01840	0.772	↓	18.57
24	neg	7.57	696.4689	M + Cl	PE (P-18: 0_13: 0)	−7.776	0.00027	0.528	↓	28.00
25	neg	7.78	762.5722	M + FA − H	PE (P-18: 0_17: 0)	9.407	0.01114	0.764	↓	13.19
26	neg	13.51	796.5545	M + FA − H	PE (P-18: 0_20: 4)	6.307	0.02060	1.223	↑	27.33
27	neg	10.93	794.5401	M + FA − H	PE (P-18: 0_20: 5)	7.929	0.00260	1.300	↑	16.75
28	neg	11.58	820.597	M + Cl	PE (P-18: 0_22: 1)	−2.904	0.00037	0.723	↓	18.92
29	neg	10.44	802.5798	M − H	PE (P-20: 0_22: 6)	5.269	0.00039	0.663	↓	9.27
30	neg	10.51	785.5129	M + Cl	PG (18: 0_16: 0)	3.202	0.00092	1.751	↑	10.77
31	neg	10.93	811.5285	M + Cl	PG (18: 1_18: 0)	3.008	0.00076	1.485	↑	16.55
32	neg	6.21	847.5457	M − H	PG (22: 6_20: 1)	−4.431	0.00073	0.573	↓	21.41
33	neg	4.35	905.5312	M + FA − H	PI (18: 2_18: 1)	−9.876	0.03147	0.751	↓	10.79
34	neg	12.43	889.5749	M − H	PI (22: 2_16: 0)	−7.040	0.00121	0.702	↓	11.41
35	neg	10.08	878.6138	M + FA − H	PS (18: 0_21: 0)	1.210	0.01828	0.700	↓	16.67
36	neg	8.56	792.5255	M − H_2_O − H	PS (18: 1_20: 3)	8.655	0.00255	1.626	↑	19.32
37	neg	9.28	826.5966	M − H_2_O − H	PS (18: 1_22: 0)	−0.113	0.00101	0.701	↓	14.02
38	neg	9.14	916.6283	M + FA − H	PS (18: 1_24: 1)	−0.160	0.00014	0.546	↓	16.38
39	neg	7.78	876.5505	M + Cl	PS (18: 2_22: 1)	−2.583	0.00258	0.641	↓	19.13
40	neg	7.84	816.5792	M − H	PS (20: 1_18: 0)	3.884	0.04284	0.717	↓	14.43
41	neg	9.28	890.5644	M + Cl	PS (20: 3_21: 0)	−4.546	0.00035	0.647	↓	22.84
42	neg	11	862.4866	M + FA − H	PS (22: 6_17: 2)	−1.232	0.00484	1.390	↑	24.58
43	neg	12.14	880.5401	M + FA − H	PS (22: 6_18: 0)	6.702	0.04395	0.844	↓	18.81
44	neg	10.93	811.4895	M + FA − H	SQDG (18: 0_12: 0)	1.591	0.02255	1.306	↑	26.20

*^a^* CerP, ceramide 1-phosphate; DG, diacylglycerol; PA, phosphatidylanisol; PC, phosphatidylcholine; PE, phosphatidylethanolamine; PG, phosphatidylglycerol; PI, phosphatidylinositol; PS, phosphatidylserine; SQDG, sulfoquinovosyl diacylglycerols. *p*-value of *t*-test (two-tailed, unequal variance) between PPMS and SPMS; FC, Fold change, SPMS versus PPMS; ↑, increased in SPMS compared to PPMS; ↓, decreased in SPMS compared to PPMS.

**Table 3:** List of common significant lipids identified in the normal-appearing white matter (NAWM) by RP-UPLC-TOF MS^E^ that differentiate control from PPMS, and controls from SPMS groups. Lipids are sorted in classes and listed alphabetically (ascending).

							*p* Value		FC		Trend		
Peak Number	MS Mode	RT	*m/z*	Adduct	Lipid Identified	Delta (ppm)	CON ^a^-PPMS	CON-SPMS	PPMS/CON	SPMS/CON	PPMS/CON	SPMS/CON	RSD% in QCs
1	pos	3.05	719.5589	M + Na	DG (20: 4_22: 2)	0.526	0.0448	0.0238	0.875	0.853	↓	↓	15.86
2	pos	8.36	704.5254	M + NH_4_	PA (18: 2_17: 0)	4.296	0.0096	0.0025	0.796	0.731	↓	↓	15.57
3	pos	6.28	738.5091	M + NH_4_	PA (20: 5_18: 1)	3.127	0.0376	0.0102	0.585	0.472	↓	↓	17.74
4	neg	12.14	752.5621	M − H_2_O − H	PE (18: 2_20: 0)	2.771	0.0003	0.0065	1.553	1.540	↑	↑	25.34
5	neg	13.86	840.5768	M + FA − H	PE (20: 4_20: 0)	0.943	0.0017	0.0020	1.306	1.315	↑	↑	17.26
6	neg	8.72	828.5276	M + Cl	PE (20: 4_20: 1)	−5.046	0.0105	0.0053	0.589	0.549	↓	↓	25.55
7	neg	10.71	882.5863	M + Cl	PE (22: 6_22:0)	9.208	0.0444	0.0164	0.671	0.588	↓	↓	24.42
8	neg	8.2	819.5428	M − H_2_O − H	PI (18:0_16:0)	4.231	0.0163	0.0079	0.621	0.583	↓	↓	21.29
9	neg	8.36	846.5546	M + FA − H	PS (18:2_19:0)	5.461	0.0102	0.0092	0.786	0.799	↓	↓	15.08
10	neg	8.85	880.5744	M + FA − H	Sulfatide (d18: 1/20: 0)	−9.753	0.0111	0.0040	0.759	0.729	↓	↓	20.69

DG, diacylglycerol; PA, phosphatidylanisol; PE, phosphatidylethanolamine; PI, phosphatidylinositol; PS, phosphatidylserine. *p*-value of *t*-test (two-tailed, unequal variance) between PPMS/SPMS and controls; FC, Fold change; ↑, increased in PPMS/SPMS compared to controls; ↓, decreased in PPMS/SPMS compared to controls. ^a^ CON, controls.

## Supplementary Materials

The following are available online at https://www.mdpi.com/2218-1989/10/9/366/s1, Figure S1: Immunohistochemistry for myelin using anti-MOG antibody (A,B) and microglia stained with anti-HLADR antibody (C,D) on frozen sections of Control (A & C) (case 39) and MS (case 275) samples. Sections are counterstained with haematoxylin (blue). Bar represents 50 µm. Figure S2A: Positive ion mode; Controls vs. PPMS. A permutation test performed with 100 random permutations on the generated OPLS-DA model showed no overfitting of the model (Q2 = (0.0, −0.557)). Figure S2B: Positive ion mode; Controls vs. SPMS. A permutation test performed with 100 random permutations on the generated OPLS-DA model showed no overfitting of the model (Q2 = (0.0, −0.517)). Figure S2C: Positive ion mode; PPMS vs. SPMS. A permutation test performed with 100 random permutations on the generated OPLS-DA model showed no overfitting of the model (Q2 = (0.0, −0.512)). Figure S3A: Negative ion mode; Controls-PPMS. A permutation test performed with 100 random permutations on the generated OPLS-DA model showed no overfitting of the model (Q2 = (0.0, −0.546)). Figure S3B: Negative ion mode; Controls-SPMS. A permutation test performed with 100 random permutations on the generated OPLS-DA model showed no overfitting of the model (Q2 = (0.0, −0.557)). Figure S3C: Negative ion mode; PPMS-SPMS. A permutation test performed with 100 random permutations on the generated OPLS-DA model showed no overfitting of the model (Q2 = (0.0, −0.512)). Table S1: Patient Demographics of control, PPMS, and SPMS groups. Samples are sorted by groups: controls (n = 8), PPMS (n = 9), SPMS (n = 7). Excel file Table S2a,b: Raw abundances of ions/features used to create the Venn diagrams in Figure 3. Table S3: Correlation analysis of lipids with sex, age, PMI.
