# Supplementary material for: Lipidomic UPLC-MS/MS Profiles of Normal-Appearing White Matter Differentiate Primary and Secondary Progressive Multiple Sclerosis
Source: Metabolites. 2020 Sep 8;10(9):366. doi: 10.3390/metabo10090366 (PMC7569864; doi:10.3390/metabo10090366)
Supplement: Supplementary file 1 [file metabolites-10-00366-s001.zip › Figure S.1_IHC image_12JULY (1) final 01 08nw.docx]

**Figure S.1**


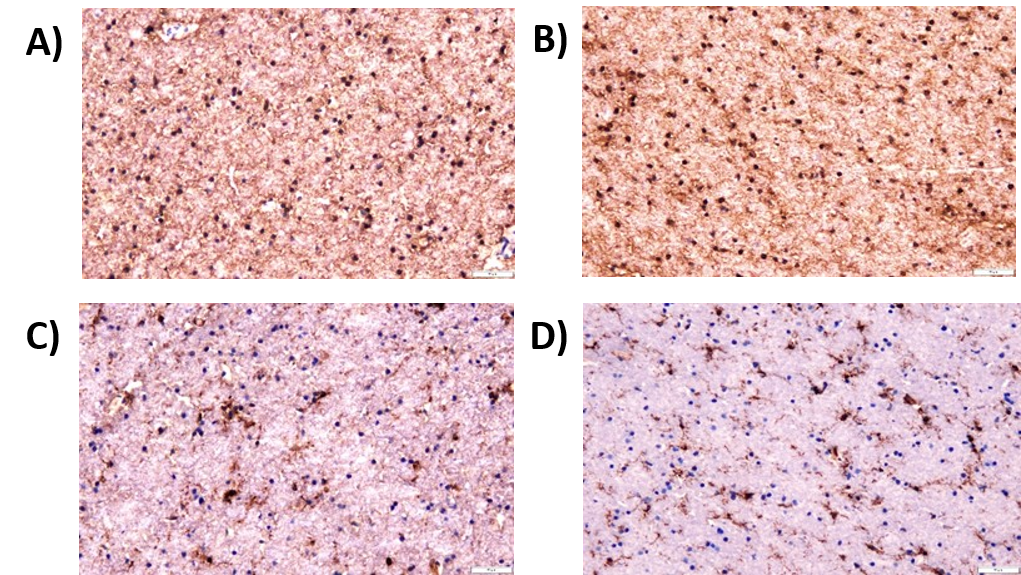


**Control MS**

**Figure S.1**. Immunohistochemistry for myelin using anti-MOG antibody (A, B) and microglia stained with anti-HLADR antibody (C, D) on frozen sections of Control (A & C) (case 39) and MS (B & D) (case 275) samples. Sections are counterstained with haematoxylin (blue). In (A) and (B) the myelin is stained brown and there is an overall even intensity of stain for both control and MS samples, indicating that there has been no loss of myelin in these samples, and for the MS case this was then classified as NAWM. In (C) and (D) microglia cells within the brain parenchyma are stained brown with antibody to HLA-DR. The microglia are evenly distributed across the sections and the microglia have extended processes (arrows) indicating resting cells and hence lack of inflammation. All control and MS samples included in the study were analysed by IHC and assessed by two independent observers (NW and PP) and assessed to be in the MS cases as NAWM. Bar represents 50µm.
