# Supplementary material for: Lipidomic UPLC-MS/MS Profiles of Normal-Appearing White Matter Differentiate Primary and Secondary Progressive Multiple Sclerosis
Source: Metabolites. 2020 Sep 8;10(9):366. doi: 10.3390/metabo10090366 (PMC7569864; doi:10.3390/metabo10090366)
Supplement: Supplementary file 1 [file metabolites-10-00366-s001.zip › Figure S.2, S.3_Permutation test of OPLS-DA models_12 JULY.docx]

**Figures S.2 & S.3**

**Figure S.2A**


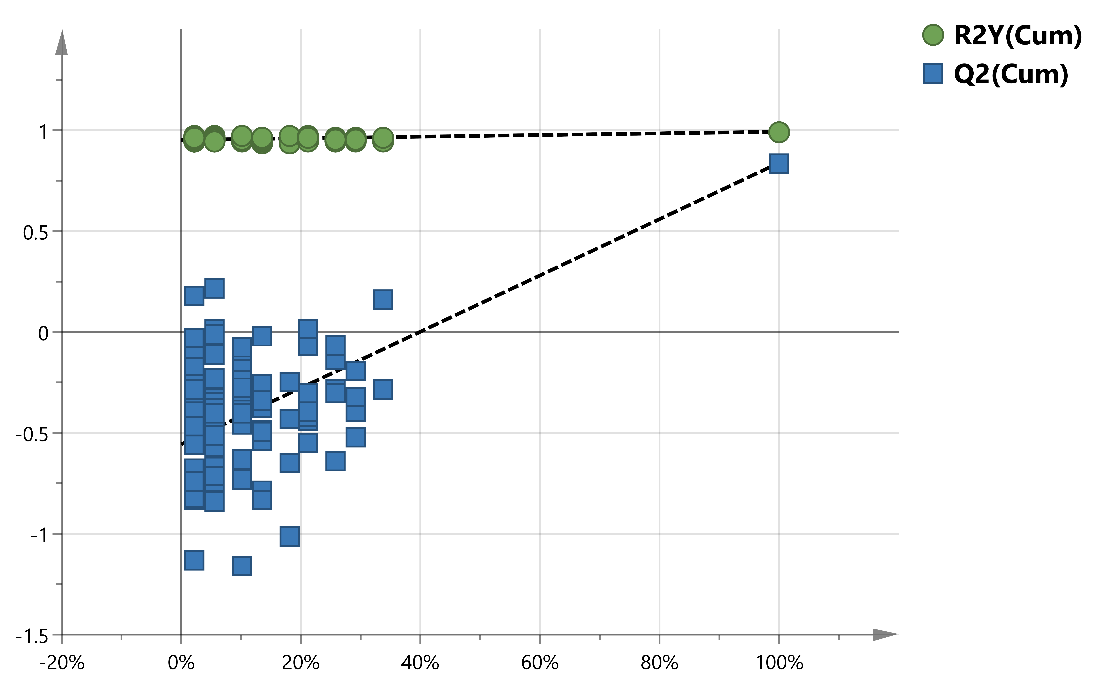


Positive ion mode; Controls vs PPMS. A permutation test performed with 100 random permutations on the generated OPLS-DA model showed no overfitting of the model (Q2 = (0.0, -0.557)).

**Figure S.2B**

**
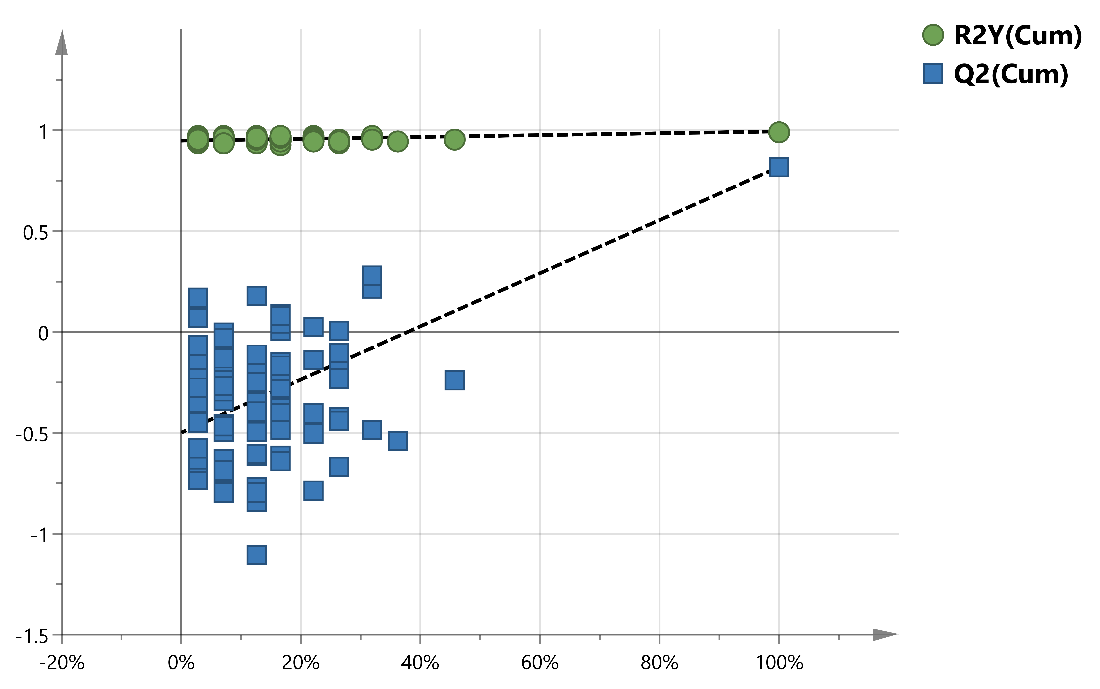
**

Positive ion mode; Controls vs SPMS. A permutation test performed with 100 random permutations on the generated OPLS-DA model showed no overfitting of the model (Q2 = (0.0, -0.517)).

**Figure S.2C**

**
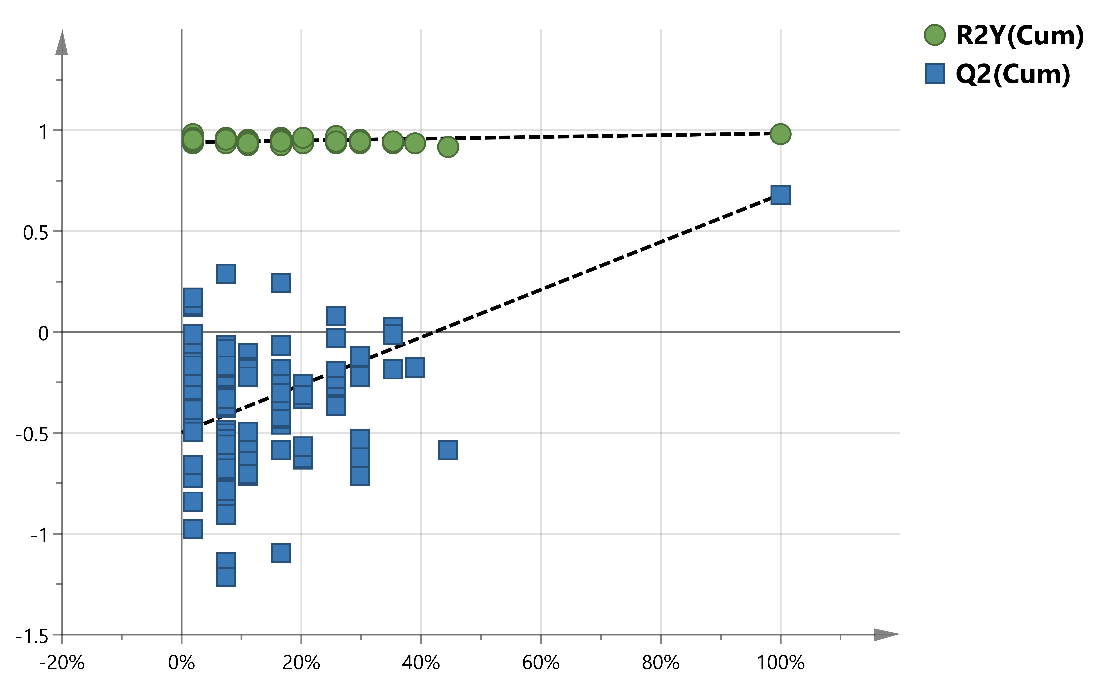
**

Positive ion mode; PPMS vs SPMS. A permutation test performed with 100 random permutations on the generated OPLS-DA model showed no overfitting of the model (Q2 = (0.0, -0.512)).

**Figure S.3A**

**
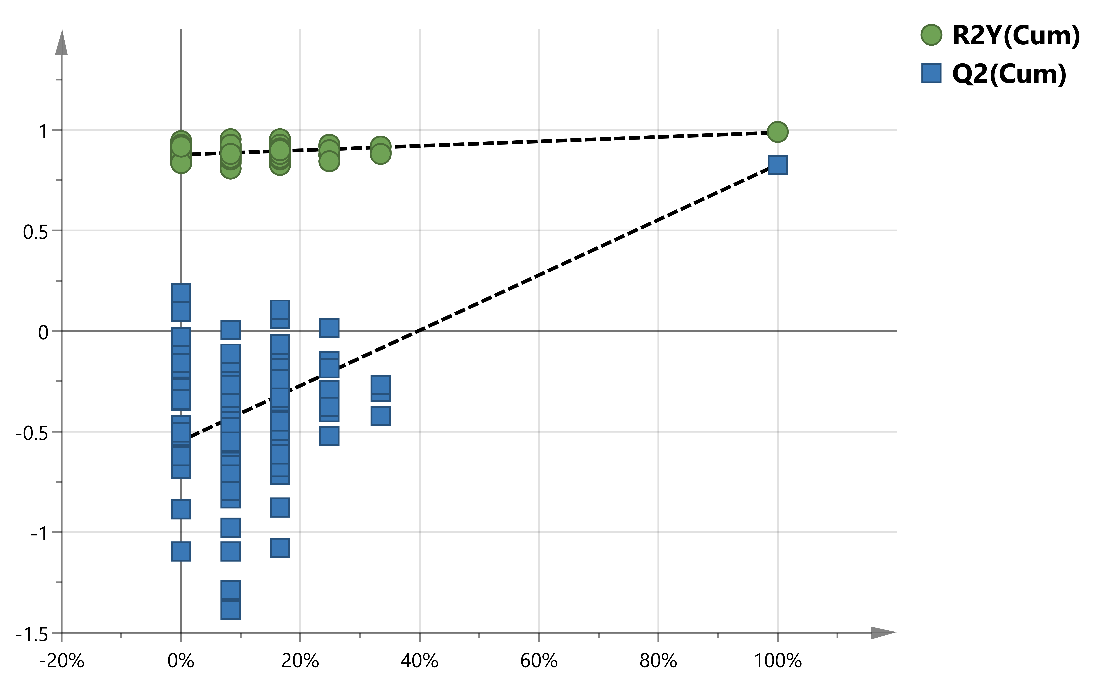
**

Negative ion mode; Controls-PPMS. A permutation test performed with 100 random permutations on the generated OPLS-DA model showed no overfitting of the model (Q2 = (0.0, -0.546)).

**Figure S.3B**

**
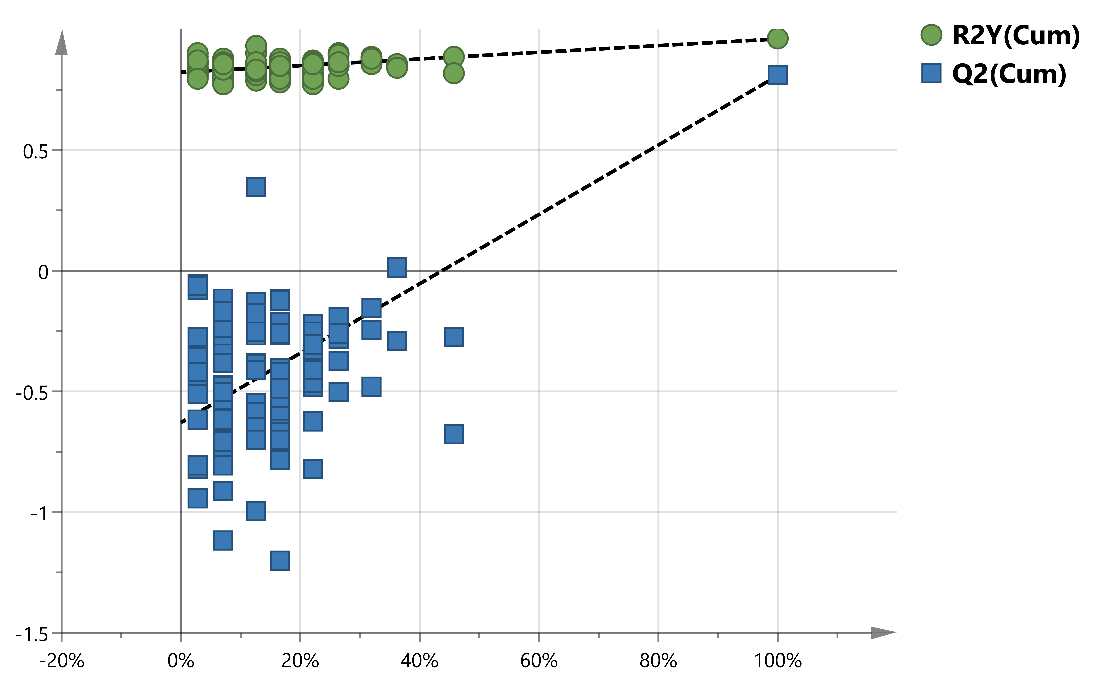
**

Negative ion mode; Controls-SPMS. A permutation test performed with 100 random permutations on the generated OPLS-DA model showed no overfitting of the model (Q2 = (0.0, -0.557)).

**Figure S.3C**


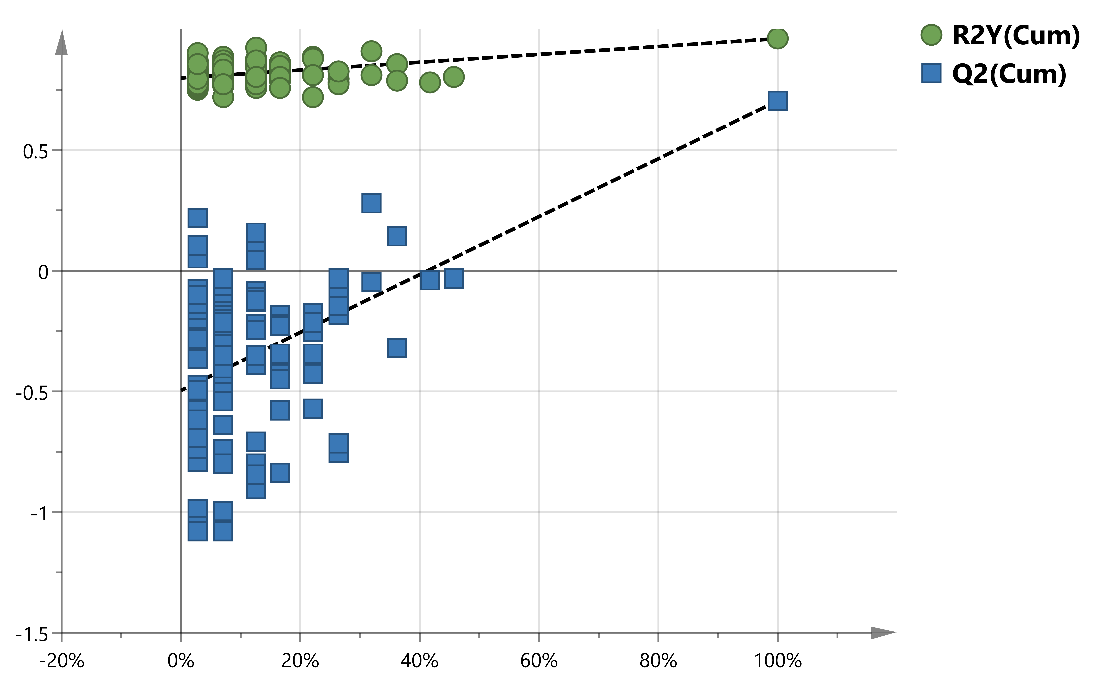


Negative ion mode; PPMS-SPMS. A permutation test performed with 100 random permutations on the generated OPLS-DA model showed no overfitting of the model (Q2 = (0.0, -0.512)).
