# Supplementary material for: Lipidomic UPLC-MS/MS Profiles of Normal-Appearing White Matter Differentiate Primary and Secondary Progressive Multiple Sclerosis
Source: Metabolites. 2020 Sep 8;10(9):366. doi: 10.3390/metabo10090366 (PMC7569864; doi:10.3390/metabo10090366)
Supplement: Supplementary file 1 [file metabolites-10-00366-s001.zip › Table S.1_Patient Demographics_12JULY.docx]

**Table S.1**

**Table S.1.** Patient Demographics of control, PPMS, and SPMS Groups. Samples are sorted by groups: controls (n=8), PPMS (n=9), SPMS (n=7)*^a^*

| Block ID | Group | Sex *^a^* | Age *^a^* | PMI *^a^* |
| --- | --- | --- | --- | --- |
| CO37_A2C3 | CONTROL | M | 84 | 5 |
| CO54_A1C4 | CONTROL | M | 66 | 16 |
| CO64_A2B2 | CONTROL | F | 63 | 21 |
| CO39_A2C4 | CONTROL | M | 82 | 21 |
| CO32_P1A1 | CONTROL | M | 88 | 22 |
| CO73_A1B1 | CONTROL | M | 71 | 29 |
| CO76_A2D3 | CONTROL | M | 87 | 31 |
| CO22_P4C3 | CONTROL | F | 69 | 33 |
|  |  |  |  |  |
| MS129_A2B2 | PPMS | F | 66 | 8 |
| MS473_A1C2 | PPMS | F | 39 | 9 |
| MS390_P2C2 | PPMS | F | 59 | 9 |
| MS325_A2C2 | PPMS | M | 51 | 13 |
| MS492_A1C4 | PPMS | F | 66 | 15 |
| MS263_A2B2 | PPMS | F | 73 | 16 |
| MS248_P2C2 | PPMS | F | 58 | 17 |
| MS102_A3C3 | PPMS | M | 73 | 20 |
| MS273_A2C3 | PPMS | M | 61 | 24 |
|  |  |  |  |  |
| MS166_P2C2 | SPMS | F | 52 | 7 |
| MS074_PID3 | SPMS | F | 64 | 7 |
| MS275_A2B2 | SPMS | F | 63 | 11 |
| MS157_A1D2 | SPMS | F | 39 | 12 |
| MS213_A1C3 | SPMS | F | 70 | 13 |
| MS168_A2B2 | SPMS | F | 88 | 22 |
| MS255_A1C1 | SPMS | M | 45 | 24 |

*^a^*M, male; F, female; age (in years); PMI (in hours); post-mortem brain tissue block ID
