# Supplementary material for: Lipidomic UPLC-MS/MS Profiles of Normal-Appearing White Matter Differentiate Primary and Secondary Progressive Multiple Sclerosis
Source: Metabolites. 2020 Sep 8;10(9):366. doi: 10.3390/metabo10090366 (PMC7569864; doi:10.3390/metabo10090366)
Supplement: Supplementary file 1 [file metabolites-10-00366-s001.zip › Table S3_Correlation lipids with sex,age, PMI_25 AUG (correct).docx]

**Table S3. Correlation of lipids with sex, age, PMI**

|  |  |  | Parameters | | |
| --- | --- | --- | --- | --- | --- |
| Number | **Ionization MS mode** | **Lipid name** | **Sex** | **Age** | **PMI** |
| 1 | neg | CerP(d18:1/20:0) |  | YES (↓) |  |
|  |  |  |  |  |  |
| 2 | pos | DG(18:0_19:0) |  | Yes (↓) |  |
| 3 | pos | DG(20:4_24:4) |  |  | Yes (↓) |
| 4 | pos | DG(20:5_18:1) |  | Yes (↑) |  |
| 5 | pos | DG(22:0_25:0) |  |  | Yes (↓) |
| 6 | pos | DG(22:6_18:0) |  | Yes (↑) |  |
|  |  |  |  |  |  |
| 7 | pos | lysoPE(22:4) |  |  | Yes (↓) |
|  |  |  |  |  |  |
| 8 | pos | PA(18:0_17:0) |  | Yes (↑) |  |
| 9 | pos | PA(18:2_18:0) |  |  | Yes (↑) |
| 10 | pos | PA(20:5_20:1) |  |  | Yes (↓) |
| 11 | pos | PA(22:6_14:0) |  | Yes (↓) |  |
| 12 | pos | PA(22:6_22:2) |  |  | Yes (↓) |
|  |  |  |  |  |  |
| 13 | pos | PC(P-16:0_17:0) |  | Yes (↑) |  |
|  |  |  |  |  |  |
| 14 | pos | PE(16:1_18:0) |  |  | Yes (↓) |
| 15 | neg | PE(18:1_20:2) |  | YES (↑) |  |
| 16 | pos | PE(18:2_18:0) |  |  | Yes (↓) |
| 17 | neg | PE(18:2_24:1) |  | YES (↓) |  |
| 18 | neg | PE(18:3_21:0) | YES (*) |  |  |
| 19 | pos | PE(20:1_17:0) |  |  | Yes (↓) |
| 20 | neg | PE(20:5_20:0) |  |  | YES (↓) |
| 21 | neg | PE(22:6_20:0) |  | YES (↑) |  |
|  |  |  |  |  |  |
| 22 | pos | PE(P-18:0_20:2) |  | Yes (↑) |  |
| 23 | neg | PE(P-18:0_22:1) | YES (*) |  |  |
| 24 | pos | PE(P-20:0_20:3) |  | Yes (↑) | Yes (↑) |
|  |  |  |  |  |  |
| 25 | neg | PG(20:4_17:0) |  | YES (↑) |  |
|  |  |  |  |  |  |
| 26 | neg | PS(18:0_24:0) |  | YES (↑) |  |
| 27 | neg | PS(18:1_17:0) |  | YES (↑) |  |
| 28 | neg | PS(18:1_21:0) |  |  | YES (↓) |
| 29 | neg | PS(18:2_18:0) |  | YES (↓) |  |
| 30 | neg | PS(20:4_20:0) |  |  | YES (↓) |
| 31 | neg | PS(22:6_22:5) |  | YES (↑) |  |
|  |  |  |  |  |  |
| 32 | pos | SM(d17:0/24:0) |  |  | Yes (↓) |

*; p<0.05
